# *Leonotis nepetifolia* Flower Bud Extract Mediated Green Synthesis of Silver Nanoparticles, Their Characterization, and *In Vitro* Evaluation of Biological Applications

**DOI:** 10.3390/ma15248990

**Published:** 2022-12-16

**Authors:** Shashiraj Kariyellappa Nagaraja, Shaik Kalimulla Niazi, Asmatanzeem Bepari, Rasha Assad Assiri, Sreenivasa Nayaka

**Affiliations:** 1 P.G. Department of Studies in Botany, Karnatak University, Dharwad 580003, Karnataka, India; 2 Department of Preparatory Health Sciences, Riyadh Elm University, Riyadh 12611, Saudi Arabia; 3 Department of Basic Medical Sciences, College of Medicine, Princess Nourah bint Abdulrahman University, Riyadh 11671, Saudi Arabia

**Keywords:** *Leonotis nepetifolia* flower buds, green synthesis, cytotoxicity, PANC-1 cell line, apoptosis, flow cytometry

## Abstract

Biosynthesis of silver nanoparticles (AgNPs) using the green matrix is an emerging trend and is considered green nanotechnology because it involves a simple, low-cost, and environmentally friendly process. The present research aimed to synthesize silver nanoparticles from a *Leonotis nepetifolia* (L.) R.Br. flower bud aqueous extract, characterize these nanoparticles, and perform *in vitro* determination of their biological applications. UV-Vis spectra were used to study the characterization of biosynthesized *L. nepetifolia*-flower-bud-mediated AgNPs (LnFb-AgNPs); an SPR absorption maximum at 418 nm confirmed the formation of LnFb-AgNPs. The presumed phytoconstituents subjected to reduction in the silver ions were revealed by FTIR analysis. XRD, TEM, EDS, TGA, and zeta potential with DLS analysis revealed the crystalline nature, particle size, elemental details, surface charge, thermal stability, and spherical shape, with an average size of 24.50 nm. In addition, the LnFb-AgNPs were also tested for antimicrobial activity and exhibited a moderate zone of inhibition against the selected pathogens. Concentration-dependent antioxidant activity was observed in the DPPH assay. Further, the cytotoxicity increased proportionate to the increasing concentration of the biosynthesized LnFb-AgNPs with a maximum effect at 200 μg/mL by showing the inhibition cell viability percentages and an IC_50_ of 35.84 μg/mL. Subsequently, the apoptotic/necrotic potential was determined using Annexin V/Propidium Iodide staining by the flow cytometry method. Significant early and late apoptosis cell populations were observed in response to the pancreatic ductal adenocarcinoma (PANC-1) cell line, as demonstrated by the obtained results. In conclusion, the study’s findings suggest that the LnFb-AgNPs could serve as remedial agents in a wide range of biomedical applications.

## 1. Introduction

Nanotechnology is an important paradigm of modern science dealing with fabrication, synthesis, characterization, strategies, and manipulation of particle shapes and sizes ranging from 1 to 100 nm and exploring their multifaceted properties [1]. Nanoparticles (NPs) possess distinctive physical, optical, electric, thermal, chemical, and biological properties, unlike their bulk materials, due to the high surface area to volume ratio (small size) and quantum effects. The properties mentioned earlier are all massively correlated with their shape, size, charge, temperature, and surface coatings, among other materials [2]. The potentiality of NPs has been explored globally in various fields, and it is easily noticeable for several applications such as cosmetics, textile, and food packaging industries, drug and gene delivery, cancer therapy, nano-medicine, bio-imaging, bio-detection of pathogens, biomedical devices, and environmental protection and energy sectors [3,4].

For the synthesis of nanoparticles, diffusion, evaporation–condensation, electrolysis, sputter deposition, pyrolysis, and plasma arcing are among the most common physical techniques. However, low production rate, costly operations, and high energy consumption are the major drawbacks of these processes [5]. In chemical synthesis techniques, the uses of toxic chemicals pose risks such as carcinogenicity and environmental toxicity. Due to the use of hazardous substances such as reductants, chemical agents, and stabilizers, the toxicity issues are quite prominent [6]. The use of toxic solvents and chemical contaminations restrict the clinical and biomedical applications of nanoparticles [7].

In recent decades, physicochemical synthesis has been supplanted by a new era of green synthesis for the development of nonhazardous nanoparticles [5] to counteract the disadvantages of conventional methods. Several components, including the reduction in derivatives or pollution, the prevention or minimization of waste, and the use of a safer (or non-toxic) solvent in addition to renewable feedstock [8,9], can explain the fundamental principles of green synthesis.

Although NPs have an optimistic future in nano biomedicine, remarkable attempts are required to comprehend the intricate mechanisms underlying their biological functions and horrific or adverse effects [10,11]. Among nanoparticles, various metal and metal oxide NPs were fabricated from copper oxide, gold, palladium, selenium, silver, zinc oxide, etc., by implementing different protocols. In addition to these metal nanoparticles, silver nanoparticles (AgNPs) have played a prominent role in wound healing due to their innate therapeutical properties [12,13].

Phyto-synthesis of AgNPs can be achieved from roots, stems, leaves, fruits, flowers and flower buds, seeds, and bark. They are inexpensive compared to microbial fabrication using bacteria, fungi, algae, etc. Microbe-mediated synthesis is relatively expensive and tedious because of the heresy of cultures [14,15]. Plant-mediated synthesis is a fast, reliable-safe, low-cost, environmentally friendly, and single-step approach. Numerous bioactive secondary metabolites, such as alkaloids, tannins, saponins, terpenoids, anthraquinones, etc., are found in plant extracts, which act as reducing, stabilizing, and capping materials in the synthesis of nanoparticles [16]. The common properties of these biosynthesized AgNPs have been investigated for use in biomedical diagnostics, antimicrobials, anticancer agents, molecular sensing, and labeling of biological systems [9].

Pancreatic cancer is the fourth leading cause of cancer deaths globally, accounting for approximately 3% of all human malignant tumors [17]. According to data, the number of people diagnosed with pancreatic cancer rose from 277,000 in 2008 to 338,000 in 2012. Approximately 266,000 people died from pancreatic cancer in 2008, and this number rose to 331,000 in 2012. It is predicted that this could increase by five times in the future [18]. Pancreatic ductal adenocarcinoma (PANC-1) is the most severe and common form of pancreatic cancer, with the current objective being to alleviate disease-related symptoms and prolong survival. Currently available therapeutic options include chemotherapy, immunotherapy, surgery, radiation, and the use of targeted drugs, all of which result in significant adverse effects in cancer patients [19].

Hence, researchers have been developing alternative drugs to expand the cancer case burden worldwide; many research communities have synthesized numerous varieties of NPs through biological approach. AgNPs play a prominent role in therapeutic applications, such as through anticancer, antidiabetic, antioxidant, antimicrobial, and antiviral activities. The existence of several phytoconstituents on the surface of AgNPs derived from the green approach is allocated to their excellent antibacterial and anticancer activities [11,20]. It has been presented that plant-mediated AgNPs have been shown to have a potentially broad spectrum of cytotoxic potential and remarkable selectivity towards the tumor cells in a concentration-dependent manner against various types of carcinoma cells. AgNPs derived from the *Tussilago farfara* flower bud extract exhibited cytotoxic activity against AGS, HT-29, and PANC-1 cell lines [21], whereas AgNPs synthesized from the *Salvia miltiorrhiza* leaf aqueous extract exhibited anticancer activity against LNCaP cell lines [22]. *Zingiber officinale* extract bio-fabricated AgNPs showed cytotoxic potential against PANC-1, AsPC-1, and MIA PaCa-2 cell lines [23]. The flower extract of *Abelmoschus esculentus*-mediated AgNPs revealed cytotoxic activity against A-549 and TERT-4 cell lines [24]. AgNPs derived from the *Datura inoxia* flower extract had a potent anti-proliferative effect on the MCF-7 cells [25].

The genus *Leonotis* has 14 species widely distributed in tropical regions and is represented by 1 species in India, *Leonotis nepetifolia* (L.) R.Br. It is an herbaceous plant or subshrub that belongs to the family Lamiaceae. Its leaves and roots are used to treat fever, cough, skin infection, stomachache, rheumatism, bronchial asthma, and kidney dysfunction in various Indian traditional systems of medicine such as Ayurveda, unani, and siddha, and are used for the same purposes in various countries such as Brazil, Canada, Madagascar, and many African nations [26]. Some compounds isolated from this herb exhibited biological activities, including antibacterial, antifungal, antidiabetic, and anti-proliferative properties [27,28]. The use of the plant’s leaves and roots is well-documented, whereas the flower and flower buds have not been extensively studied.

In the current research work, we conceptualized to synthesize silver nanoparticles from the *L. nepetifolia* flower bud extract and evaluate the characterizations, antioxidant efficacy, antimicrobial potential, and *in vitro* cytotoxicity on a pancreatic ductal adenocarcinoma (PANC-1) cell line of these nanoparticles, as well as apoptotic studies through flow cytometry.

## 2. Materials and Methods

### 2.1. Collection of Plant Material and Chemicals

*Leonotis nepetifolia* (L.) R.Br. flower buds were collected from September to December from the surroundings of Karnatak University campus, Dharwad (15°43′59.6″ N 74°98′26.8″ E), and comparing the specimens to the herbarium collection of animals and plants at Karnatak University’s departmental herbarium museum in Dharwad, Karnataka, India, confirmed the specimens’ authenticity. The chemicals and reagents, silver nitrate (AgNO_3_), dimethyl sulfoxide (DMSO), 2,2-diphenyl-1-picrylhydrazyl (DPPH), α-naphthol, methanol, ascorbic acid (Vitamin C), nutrient broth, agar, standard antibiotics (streptomycin and nystatin), 3-(4,5-dimethylthiazol-2-yl)-2,5-diphenyltetrazolium bromide (MTT), Dulbecco’s modified eagle’s medium (DMEM), fetal bovine serum (FBS), D-PBS buffer, and FITC Annexin V/Propidium Iodide (PI), were of AR grade and procured from SD-fine chemicals and Hi-media Pvt. Ltd. (Mumbai, India). Pathogenic strains *S. aureus* (MTCC 6908), *B. subtilis* (MTCC 6633), *E. coli* (MTCC 40), *P. aeruginosa* (MTCC 9027), *C. glabrata* (MTCC 3019), and *C. albicans* (MTCC 227) were procured from IMTECH, Chandigarh, India. The pancreatic ductal adenocarcinoma (PANC-1) cell line was obtained from NCCS, Pune, India.

### 2.2. Preparation of Flower Bud Extract

**Figure 1:**
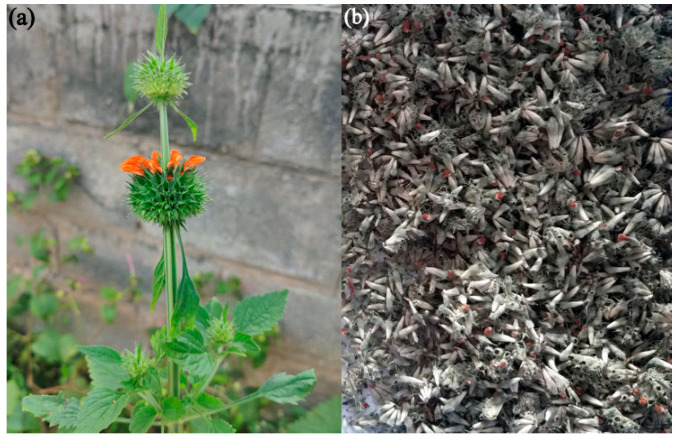
(**a**) Plant of *L. nepetifolia* with flowering twig, and (**b**) shade-dried flower buds.

The healthy unopened flower buds (Figure 1a,b) were collected and washed thoroughly with tap water, later with demineralized water, and dried in the shade. Then, they were powdered using an electric blender and stored in sterile polythene bags. Finely powdered, approximately weighed (10 g), and minced with 100 mL of Milli-Q water, the suspension was boiled for 1 h at 60 °C, cooled down, and filtered using Whatman No. 1 filter paper. The obtained filtrate was kept at 4 °C for further investigations [29].

### 2.3. Biosynthesis of AgNPs

**Figure 2:**
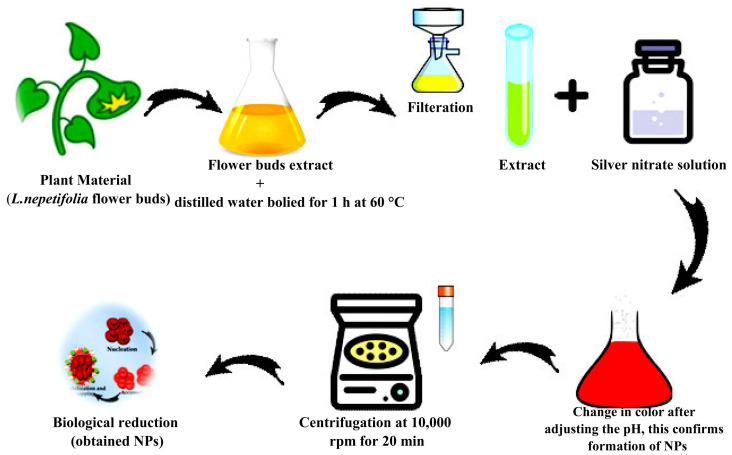
Schematic representation of biosynthesis of LnFb-AgNPs.

For biosynthesis of AgNPs, 1 mM silver nitrate (AgNO_3_) was prepared by dissolving 0.169 g in 1000 mL of deionized water. The obtained flower bud extract and AgNO_3_ solution was mixed at a ratio of 1:4 (*v*/*v*) in a 1000 mL Erlenmeyer flask for synthesis optimization (the pH of which was set to 9). The final solution was incubated for 24 h at room temperature in a dark chamber. The reduction process gradually transformed the orange solution into a dark brownish color. Thus, the change in color affirmed the accomplishment of AgNPs. Further, LnFb-AgNPs were obtained by centrifuging the biosynthesized AgNPs at 10,000 rpm for about 20 min. The collected LnFb-AgNPs were then oven-dried and stored for later experimental analysis [30]. The biosynthesis process is schematically represented in Figure 2.

### 2.4. Characterizations of Biosynthesized LnFb-AgNPs

Following the synthesis and refinement of LnFb-AgNPs, their size, shape, and morphological characteristics were confirmed using several experimental techniques.

#### 2.4.1. UV-Visible Spectrophotometric Analysis

The UV-visible spectra of biosynthesized nanoparticles were obtained by configuring a double-beam UV-visible spectrophotometer (METASH UV-9600A, Shanghai, China) with a 1 nm resolution, placing the test solution in a quartz cuvette, and analyzing its optical density at wavelengths between 300 and 600 nm. The graph was constructed by plotting wavelength (X-axis) versus absorbance (Y-axis) [31].

#### 2.4.2. Fourier Transform Infrared (FT-IR) Spectroscopy Analysis

To investigate the bioactive compounds on the synthesized LnFb-AgNPs and the *Leonotis nepetifolia* flower bud extract, Fourier transform infrared (FTIR) spectroscopy analysis was performed. Using an FTIR spectrophotometer (NICOLET 6700, Thermo Fisher Scientific, Waltham, MA, USA), the FTIR spectra of *L. nepetifolia* flower bud extract and LnFb-AgNPs in KBr pellets were measured between 400 and 4000 cm^−1^. Briefly, the *L. nepetifolia* flower bud extract and dried powder form of biosynthesized LnFb-AgNPs from the flower bud extract were combined with potassium bromide (Kbr) to produce a pellet, which was then examined for the presence of IR spectral bands. To decipher the functional groups present in the sample, spectral data between 400 and 4000 cm^−1^ in resolution were collected [31].

#### 2.4.3. X-ray Diffraction (XRD) Analysis

The dried powder form of biosynthesized LnFb-AgNPs was analyzed for XRD to determine the grain size and crystallinity by placing the AgNPs in a sample holder and then placing that in an X-ray diffractometer (Rigaku Miniflex 600, Smart-Lab SE, Tokyo, Japan) to record the spectral patterns by employing a current of 30 mA with Cu Kα radiation with an angle of 2θ ranging from 30° to 90° operating at 40 kV [32].

#### 2.4.4. Scanning Electron Microscopy (SEM) Analysis and Energy Dispersive X-ray Spectroscopy (EDS) Analysis

Using scanning electron microscopy (SEM) coupled with an energy dispersive X-ray (EDS) instrument, the topology and elemental compositions of LnFb-AgNPs were determined using (JEOL, JSM IT 500LA, Peabody, MA, USA). Briefly, LnFb-AgNPs were placed on the stub using carbon tape, then fixed and covered with gold using sputtering, and the loaded stub was placed in the instrument chamber for analysis [30].

#### 2.4.5. Transmission Electron Microscopy (TEM) Analysis

To determine the size and structure of LnFb-AgNPs, images from a 300 kV transmission electron microscope (TEM) (FEI, TECNAI G2, F30, Beijing, China) were acquired. Before analysis, approximately 5 µL of LnFb-AgNPs were deposited on the TEM copper grid, followed by the application of a carbon tape coating and drying in desiccation for 48 h [31].

#### 2.4.6. Zeta Potential Analysis and Dynamic Light Scattering (DLS) Analysis

In order to determine the surface charge and stability in a solution, LnFb-AgNPs were centrifuged for 20 min at 8000 rpm to collect the supernatant, then diluted with MilliQ water and ultrasonified for 15 min. Subsequently, the solution was analyzed with a zeta analyzer. Using nano-analyzer equipment (Horiba scientific nanoparticle analyzer SZ-100, Kyoto, Japan), a DLS analysis was performed on the supernatant solution to determine the dispersal pattern, size, and surface charge of silver nanoparticles [30,31].

#### 2.4.7. Thermo Gravimetric (TGA) Analysis

To investigate the thermal behavior and nature of biosynthesized LnFb-AgNPs, thermo gravimetric analysis (TGA) was performed; a known quantity of weighed sample was placed in a furnace, and the heating temperature was increased gradually by passing inert gas over the sample. TGA analysis was conducted with an increasing heat rate of 10 °C per min from room temperature (RT-27 °C) to 600 °C using a TA instrument (SDT Q 600, New Castle, DE, USA) [32,33].

### 2.5. Antioxidant Activity of Biosynthesized LnFb-AgNPs

With some minor modifications described by Singh et al. (2021), we used the DPPH free radical assay to evaluate the antioxidant activity of the *L. nepetifolia* flower bud extract and biosynthesized LnFb-AgNPs [34]. In brief, samples of the same volume measured (0.2 mL) at different concentrations (100 to 1000 μg/mL) were added to 2 mL of DPPH solution (0.003% DPPH prepared in methanol), with ascorbic acid serving as a standard. After that, the tubes were stored in the dark for 30 min at room temperature. After incubation, a UV-Visible spectrophotometer was used to measure the absorbance of the solutions at 517 nm (METASH UV-9600A, Shanghai, China). A decrease in absorbance indicated a reduction in DPPH free radicals in the solution, calculated by the following formulae.

$$\mathrm{Scavenging}\;\mathrm{activity}\;(\%)=\frac{A_{0}-A_{1}}{A_{0}}\times 100$$

where A_0_ = absorbance of DPPH and A_1_ = absorbance of the experimental sample.

### 2.6. Antimicrobial Activity of Biosynthesized LnFb-AgNPs

Using the agar well diffusion method, the antimicrobial activity of biosynthesized LnFb-AgNPs was evaluated against selected pathogenic bacteria and fungi. Four bacterial strains were selected: two Gram-negative, *E. coli* (MTCC 40) and *P. aeruginosa* (MTCC 9027), two Gram-positive, *S. aureus* (MTCC 6908) and *B. subtilis* (MTCC 6633), and two fungal strains, *C. albicans* (MTCC 227) and *C. glabrata* (MTCC 3019). Pure cultures of pathogens were sub-cultured on their respective agar medium. The agar plates were swabbed with a bacteria and fungi suspension using cotton swabs. A gel-hole punch was used to bore 6 mm wells on 4 mm thick agar plates (pH was set to 7.4). Subsequently, four different concentrations (25 to 100 µg/µL) of LnFb-AgNPs were laden inside the wells. As positive controls, streptomycin and nystatin were used, and then culture plates were incubated at 37 °C overnight. In accordance with the standard antibiotic zone of inhibition chart, the diameter of a zone of inhibition (around the wells, mm) was measured [35].

### 2.7. In Vitro Anticancer Activity of Biosynthesized LnFb-AgNPs

Pancreatic ductal adenocarcinoma (PANC-1, cell line obtained from NCCS, Pune, India) cells were cultured for 24 h at 37 °C, 95% humidity, and 5% CO_2_ atmospheric condition to promote cell proliferation on DMEM supplemented with 10% FBS. After incubation, the cells were seeded at a density of 20,000 cells/well in 200 µL of medium in a 96-well plate. In contrast, biosynthesized LnFb-AgNPs (12.5–200 µg/mL) were added to wells with PANC-1 cells and incubated at 37 °C for 24 h. The assay included a positive control with doxorubicin (4 µM/mL), and cells lacking LnFb-AgNPs were used as a negative control. Then, MTT solution was prepared in a growth medium in order to determine the viability of the cells. A freshly prepared MTT solution (200 µL, 0.5 mg/mL) was added to each well containing cell culture and incubated at 37 °C (4 to 5 h). Following post-incubation, formazan crystals were minced in 100 µL of DMSO, and viable cells were measured at 570 nm using a microplate reader (ELX-800, BioTek, Winooski, VT, USA) [36,37]. Final results were expressed as an IC_50_ value.

### 2.8. Annexin V/PI FITC Assay for Apoptotic Analysis

Analyses of apoptotic/necrosis cells were evaluated by staining with Annexin V-FITC/PI assay (Annexin V-FITC apoptosis kit, BD Biosciences, Franklin Lakes, NJ, USA) and the results were determined according to the manufacturer’s protocol. PANC-1 (pancreatic ductal adenocarcinoma) cells were laden in a 6-well plate (at a density of 0.5 × 10^6^ cells/2 mL) and incubated overnight at 37 °C in a CO_2_ incubator. The cells were then treated with the IC_50_ concentration of LnFb-AgNPs (35.84 μg/mL) following incubation. The cells were accumulated by EDTA-trypsinization, washed twice with PBS solution, and then 5 μL of Annexin V binding buffer was added. The cells were then vortexed gently and incubated for 15 min at room temperature (27 °C) in the dark. Later, 5 μL of Propidium Iodide and 400 μL of 1X binding buffer were added to each tube and delicately vortexed. In addition, a flow cytometer (BD FACS Calibur) was used to analyze the test samples in accordance with the method developed by O’Brien and Bolton (1995). The software BD Cell Quest Pro Ver.6.0 was used to calculate the results of the experiment [38].

### 2.9. Statistical Analysis

All analyses were carried out in three sets, and the data were presented as mean standard deviation (SD). SPSS v17 and Origin 2022b software were used to perform the statistical analysis.

## 3. Results and Discussion

### 3.1. Phyto-Synthesis of LnFb-AgNPs and Their Characterizations

**Figure 3:**
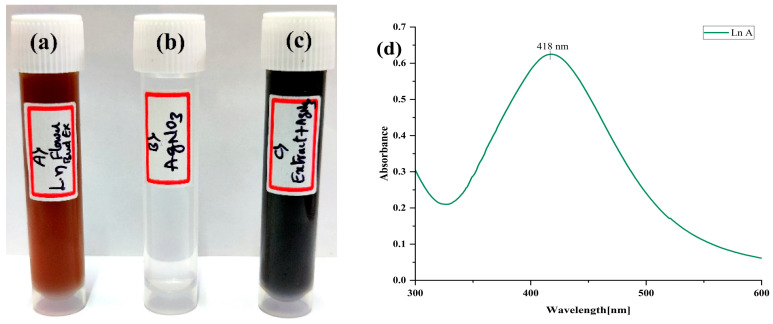
Change in color of the solution: (**a**) flower bud extract; (**b**) AgNO_3_ solution; (**c**) flower bud extract + AgNO_3_; (**d**) UV-Vis absorption spectrum of biosynthesized LnFb-AgNPs from *L. nepetifolia* flower bud extract.

In the current study, we synthesized the AgNPs from an aqueous extract of *L. nepetifolia* flower buds. A change in extract color confirmed the synthesis following the addition of AgNO_3_ (1 mM, 1:4); after 24 h of incubation at RT (27 °C), at pH 9.0, the suspension turned from orange to dark brown (Figure 3a–c). In the range of 300 to 600 nm, the characteristic SPR absorption spectrum of the synthesized LnFb-AgNPs was observed at 418 nm (Figure 3d), confirming the synthesis of LnFb-AgNPs. The synthesis was affirmed by the transformation of the solution’s color from orange to a dark brownish color, a phenomenon attributed to SPR. The absorption spectrum peaks of the biosynthesized LnFb-AgNPs occurred at 418 nm with a high absorbance value, which is characteristic of silver nanoparticles. In general, distinctive AgNPs show characteristic surface plasmon resonance at wavelengths ranging from 400 to 450 nm; the same was also observed in the current investigation. The spectral absorption peaks rise as extract concentration and time increase. Therefore, flower bud extract concentrations provide the time-dependent optimal amount of phytoconstituents required to reduce silver ions (Ag^+^) into AgNPs [39,40].

The pH value always plays a crucial role in a reaction mixture. The color of the reaction mixture, the intensity of the SPR peak, and the size and shape of the nanoparticles were found to be pH-dependent. The absorbance peak of the LnFb-AgNPs increases with the pH of the suspension, with maximum production occurring at pH 9 [41]. Similar outcomes were reported by Rajesh et al. (2016), where *Couroupita guianensis* Aubl. flower bud extract mediated AgNPs showed a change in color from yellowish to brown at 420 nm [42]. Pereira et al. (2020) reported that silver nanoparticles synthesized using different parts of *Handroanthus heptaphyllus* displayed comparable SPR peaks at 450 nm at pH 8–10 [43].

### 3.2. FTIR Spectroscopy Analysis

**Figure 4:**
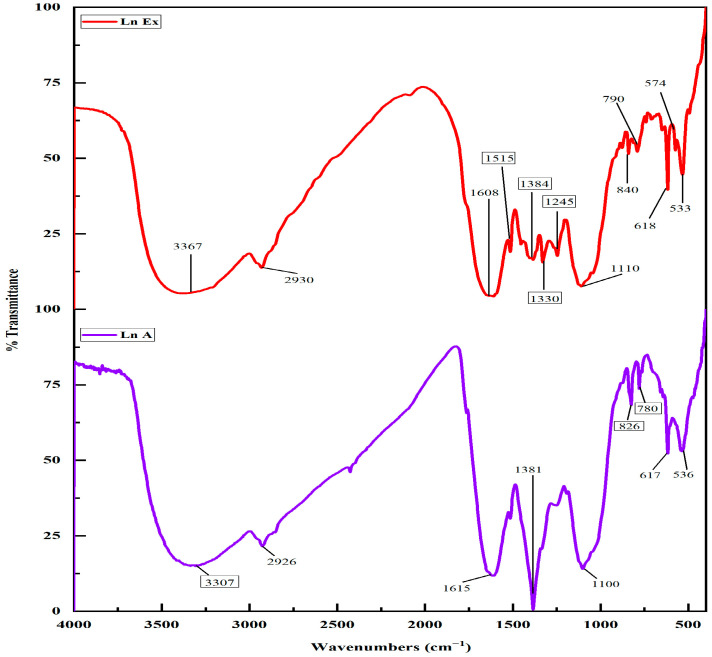
FTIR spectrum of flower bud extract and biosynthesized LnFb-AgNPs.

Diversified secondary metabolites stabilize silver nanoparticles synthesized through biomimetic methods via a molecular level of interaction with metallic surfaces. The nature of the functional groups’ interplay between synthesized materials was studied using FTIR analysis. The FTIR spectra of the *L. nepetifolia* flower bud extract exhibited peaks at 533, 574, 616, 790, 840, 1110, 1245, 1330, 1384, 1515, 1608, 2930, and 3367 cm^−1^, whereas the LnFb-AgNPs showed major peaks at 536, 617, 780, 826, 110, 1384, 1615, 2926, and 3307 cm^−1^. As a result of bio-reduction, the FTIR spectra of the extract and the LnFb-AgNPs exhibited minute shifts in peak positions. As shown, the spectrum of the *L. nepetifolia* flower bud extract differed little from the spectrum of the LnFb-AgNPs (Figure 4). The results obtained in the flower bud extract exhibited a peak at 574 cm^−1^, a weak peak for a (C-Br) stretching halo compound, and 840 cm^−1^ for (C-C) bending alkene. In the FTIR spectrum of the LnFb-AgNPs, a medium peak for (C-O) aliphatic ether was shifted to lower wave numbers at 536 cm^−1^ (C-Br), 826 cm^−1^ (C-Cl), and 1100 cm^−1^ (C-O), respectively. In contrast to the normal weak peak at 1515 cm^−1^ for the (N-O) stretching nitro compound and the broad peak at 2930 cm^−1^ for (C-H) stretching alkane, these peaks have shifted to higher wave numbers, i.e., 1615 cm^−1^ (C=C) and 2976 cm^−1^ (O-H). In the FTIR spectrum of the LnFb-AgNPs, the peak at 1245 cm^−1^ for (C-N) stretching of amine disappeared. The analysis of the FTIR spectra of the flower bud extract and LnFb-AgNPs revealed the presence of various functional groups, including phenolic/hydroxyl, alkenes, alkanes, and amines, which are involved in the interactions between bio-molecules and metal particles. Due to its electron-donating property, the flower bud extract contains several phytoconstituents that may facilitate the reduction and stabilization of silver ions (Ag^+^) to (Ag^0^) and the formation of AgNPs [44,45]. In a previous study, the mechanism of adsorption and capping of silver nanoparticles by plant extracts can be explained by the interactions and coordination of different carbonyl bonds and adjacent electron transfer to AgNPs [46]. Similarly, Algebaly et al. (2020) reported that the phenolic compounds present in the aqueous extracts act as a reducing, capping, and stabilizing agent to convert silver nitrate into AgNPs [47].

### 3.3. XRD Analysis

**Figure 5:**
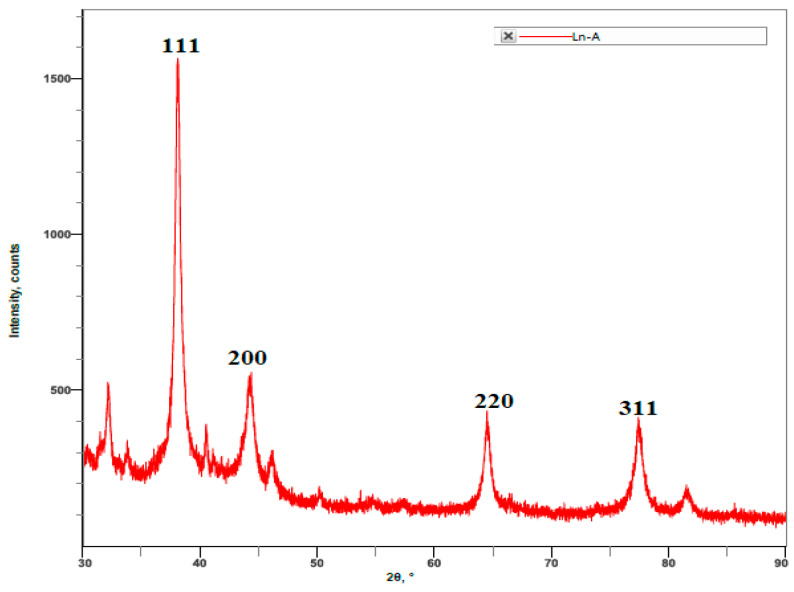
XRD spectra of biosynthesized LnFb-AgNPs from *L. nepetifolia* flower bud extract.

The XRD patterns of the biosynthesized LnFb-AgNPs are displayed in Figure 5. Bragg reflection peaks were found in all patterns of synthesized silver nanoparticles, located at 2θ values of 38.10°, 44.22°, 64.44°, and 77.37°, which correspond to the (111), (200), (220), and (311) crystallographic planes of Ag’s face-centered cubic (fcc) structure of AgNPs. The obtained results were confirmed after matching with the standard silver card JCPDS reference code 04-0783. The crystalline nature of the biosynthesized LnFb-AgNPs was confirmed by the sharp diffraction peaks. The observed peaks in the XRD analysis clearly indicated that the silver ions (Ag^+^) had completely reduced to (Ag^0^) by stabilizing and reducing compounds in the aqueous extracts. The XRD analysis also exhibited some extra peaks that were unassigned. The presence of bioorganic phases on the particle surfaces accounted for the additional unassigned peaks [48]. This result can be compared to the findings of Ajitha et al. (2019), in which AgNPs synthesized using *Syzygium aromaticum* (clove) extract displayed the same patterns as AgNPs produced under optimal conditions. The AgNPs produced four dominant diffraction peaks positioned at 2θ, 38.0°, 44.1°, 64.4°, and 77.4°, which were correlated with the lattice planes of the face-centered cubic (fcc) structure and crystalline nature of metallic silver [49].

### 3.4. SEM and EDS Analysis

**Figure 6:**
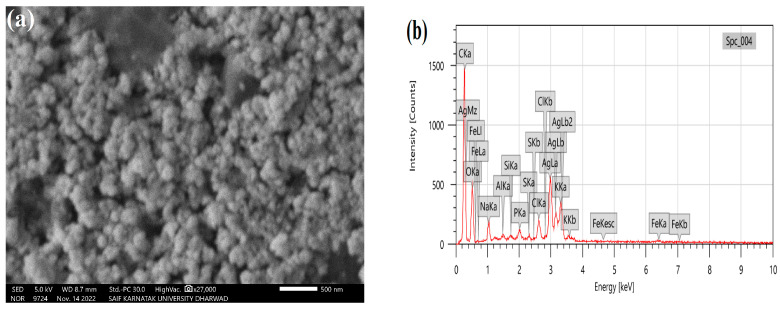
(**a**) SEM micrograph and (**b**) EDS spectrum of biosynthesized LnFb-AgNPs from *L. nepetifolia* flower bud extract.

The LnFb-AgNPs exhibited a granular morphology, as shown in Figure 6a, which was captured by SEM. The image revealed that the NPs were polydispersed and spherical, with minor agglomeration. An EDS analysis at 5 keV was employed to assess the purity and different elemental components. In Figure 6b, the EDS spectrum exhibited an elemental peak signal from silver metal at 3.09 keV and the presence of 35.34% of a sliver; typically, metallic AgNPs display an optical absorption peak at approximately 3 keV due to their SPR. Other than silver metal, a few peaks are attributed to the presence of conjugated bio-molecules over the surface AgNPs or chlorine on the glass slide used while preparing the samples for analysis. The biosynthesis of AgNPs using the extract from the aerial parts of plant formed a product with many dimensions; due to the variable values of NPs, they are distinct, with considerable alteration due to the optical and electronic properties of metallic NPs [25,50]. Similar results were obtained by Allafchian et al. (2018), who explained that the synthesis of NPs using a *Glaucium corniculatum* (L.) curtis extract yielded spherical, polydispersed, and agglomeration-free nanoparticles with a single peak at 3 keV, confirming the presence of silver metal [51].

### 3.5. TEM Analysis

**Figure 7:**
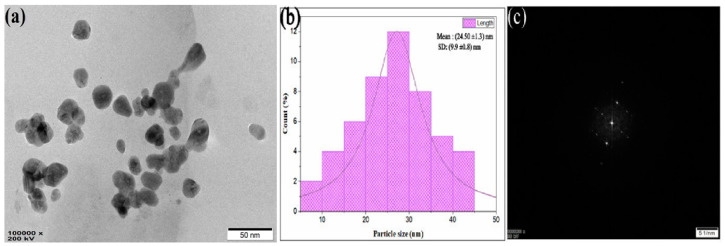
(**a**) TEM image, (**b**) histogram showing particle size distribution, and (**c**) SAED pattern image of biosynthesized LnFb-AgNPs from *L. nepetifolia* flower bud extract.

The TEM analysis revealed the NPs’ size, shape, texture, surface morphology, and distribution. Figure 7a,b are TEM micrographs of the LnFb-AgNPs, demonstrating the formation of polydisperse, spherical AgNPs with minor aggregation. The size of the LnFb-AgNPs, as depicted by the TEM image, ranged from 10–45 nm; the TEM micrographs disclosed the average particle size to be 24.50 nm. In the focusing zone, the SAED pattern (Figure 7c) revealed the crystalline nature and distribution of LnFb-AgNPs. A crystalline analysis of LnFb-AgNPs at 5 1/nm resolution revealed crystal lattice fringes with a d-spacing value of 0.207 nm. However, LnFb-AgNPs were evenly spread over the surface with minor agglomeration. This is in accordance with the result of Rajesh et al. (2016), who determined that *Couroupita guianensis* Aubl. flower bud extract mediated AgNPs had sizes of synthesized NPs that ranged from 5 to 40 nm, with an average of approximately 17 nm [42]. Similarly, the NPs synthesized using a clove bud extract had a polydispersed nature and nanoparticle sizes ranging from 10 to 50 nm without agglomeration [52].

### 3.6. Zeta Potential and DLS Analysis

**Figure 8:**
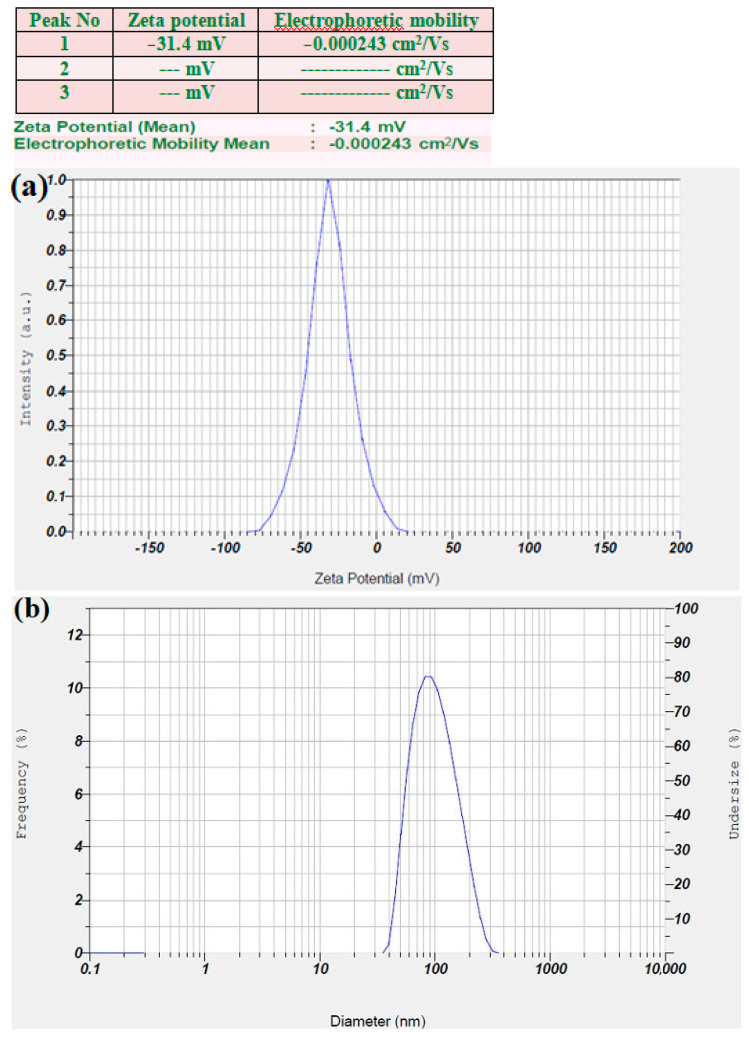
(**a**) Zeta potential analysis graph and (**b**) dynamic light scattering analysis of biosynthesized LnFb-AgNPs from *L. nepetifolia* flower bud extract.

The zeta potential of the prepared LnFb-AgNPs was dispersed in an aqueous colloidal solution at ambient temperature and exhibited a negative value of −31.4 mV (Figure 8a), indicating a high negative surface charge. The stability of the LnFb-AgNPs is a result of their relative surface charges, which prevents agglomeration. Therefore, the LnFb-AgNPs in the synthesized colloidal medium are exceptionally stable and evenly dispersed. The negative zeta potential value may be attributable to the capping of phyto-organic components present in the *L. nepetifolia* flower bud extract, as well as the electrostatic stabilization of LnFb-AgNPs in the colloidal medium. The negative values indicate that the NPs are stable. In a DLS analysis, nanoparticle hydrodynamic size is measured by scattered light from the nanocore and phytomolecule cloud as a function of time. While TEM measures the diameter of individual particles, consequently, the nanoparticle size estimated by DLS analysis is slightly larger than the actual size determined by TEM. The average size of LnFb-AgNPs is 98.5 nm and depicted in Figure 8b, which is significantly bigger than the TEM findings. Similarly, El-Aswar et al. (2019) reported that the zeta potential of the NPs synthesized from a *Haplophyllum tuberculatum* extract was −42 mV, and the nanoparticle hydrodynamic size measured by scattered light was 86.3 nm. Zeta potential values greater than −30 mV or greater than +30 mV are typically considered stable; this is due to the negatively charged electrostatic repulsive forces, which likely create a greater energy barrier to preserve the silver nanoparticles in the colloidal solution without coagulation [53]. The greater negative surface charge value, according to Ardestani et al. (2016), is due to the constructive functional bioactive phytoconstituents as a capping agent in the plant extract [54].

### 3.7. TGA Analysis

**Figure 9:**
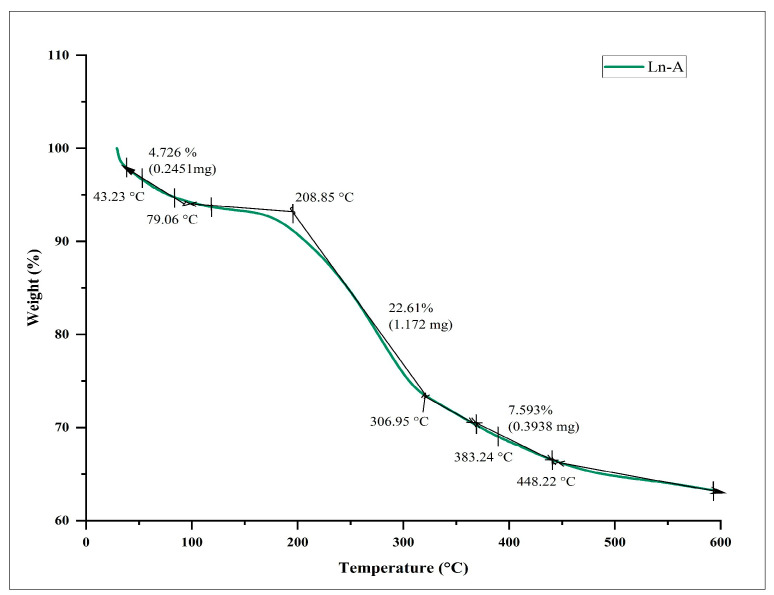
Thermo gravimetric analysis curve of biosynthesized LnFb-AgNPs from *L. nepetifolia* flower bud extract.

Using thermo gravimetric analysis, the thermal behavior and stability of biosynthesized LnFb-AgNPs were evaluated. The TGA curve (Figure 9) reveals that the biosynthesized LnFb-AgNPs were extremely steady and stable at temperatures ranging from 27 °C to 600 °C, with minimal weight loss. Between 43 °C and 208 °C, 209 °C and 307 °C, and 308 °C and 448 °C, the LnFb-AgNPs exhibited three significant weight losses of 4.72%, 22.61%, and 7.59%, for a total weight loss of approximately 35%. The initial weight loss was caused by the evaporation of the AgNPs’ moisture content. Similarly, the desorption of organic bioactive phytochemicals, which act as conjugated molecules on the surface of silver nanoparticles, was primarily responsible for the two subsequent degradations. It indicates that bioactive phytoconstituents present in nanoparticles are accountable for their reduction and stabilization [55]. This result can be compared to the findings of Moteriya and Chanda (2017), who discovered that the *Caesalpinia pulcherrima*-flower-extract-mediated NPs exhibited a constant weight loss in temperatures from 0–800 °C, and the total weight loss up to 800 °C for the synthesized AgNPs is approximately 71.68% [56].

### 3.8. Antioxidant Activity

**Figure 10:**
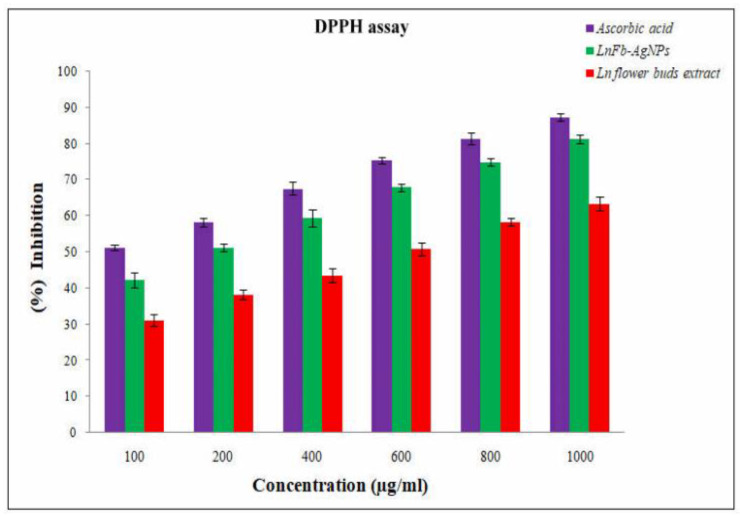
Graph showing antioxidant activity of biosynthesized LnFb-AgNPs from *L. nepetifolia* flower bud extract.

The antioxidant activity of the flower bud aqueous extract and biosynthesized LnFb-AgNPs was evaluated using the DPPH free radical scavenging assay. DPPH is a stable compound and conventional free radical that can be reduced by accepting hydrogen or an electron from ions that donate them. The antioxidant-reducing potentiality of the biosynthesized AgNPs was determined by visualizing the change in color formation. The DPPH assay demonstrated that the LnFb-AgNPs inhibited oxidative stress more effectively than flower buds; the aqueous extract is depicted in (Figure 10) and ascorbic acid served as the standard. The scavenging activity of the LnFb-AgNPs was found to increase with increasing concentration. The LnFb-AgNPs were shown in serial concentrations of 100, 200, 400, 600, 800, and 1000 µg/mL, with inhibition percentages of 42.3 ± 2.02%, 51.3 ±1.04%, 59.19 ± 1.95%, 67.9 ± 1.01%, 75 ± 1%, and 81 ± 1.12%, respectively. The antioxidant activity of biosynthesized silver nanoparticles could be associated with the plant-derived functional groups attached to them. The DPPH free radical scavenging activity of the LnFb-AgNPs was found to be dose-dependent. To investigate the scavenging activity of the flower bud extract, the ability of DPPH to readily accept a H^+^ or e^−^ from an antioxidant moiety under stable conditions was analyzed. Compared to the flower bud extract, the LnFb-AgNPs exhibited moderate reducing power. This activity is due to the presence of phenolic compounds from the extract as capping agents and stabilizers on the surface of the LnFb-AgNPs. The antioxidant capacity demonstrates the ability of silver nanoparticles to transfer electrons and neutralize reactive DPPH radicals in the reaction solution [57,58]. The results of our AgNPs derived from the *L. nepetifolia* flower bud extract demonstrate their significant antioxidant potential when compared to previously published data where AgNPs where synthesized from aqueous corn leaves. When the concentration of the AgNPs was increased, the extract showed a dose-dependent reduction in free radicals that was proportional to the dose. At 100 µg/mL, it displayed a moderate DPPH radical scavenging activity of 36.3%, while at 1000 µg/mL, it displayed 89.01% [59].

### 3.9. Antimicrobial Activity

**Figure 11:**
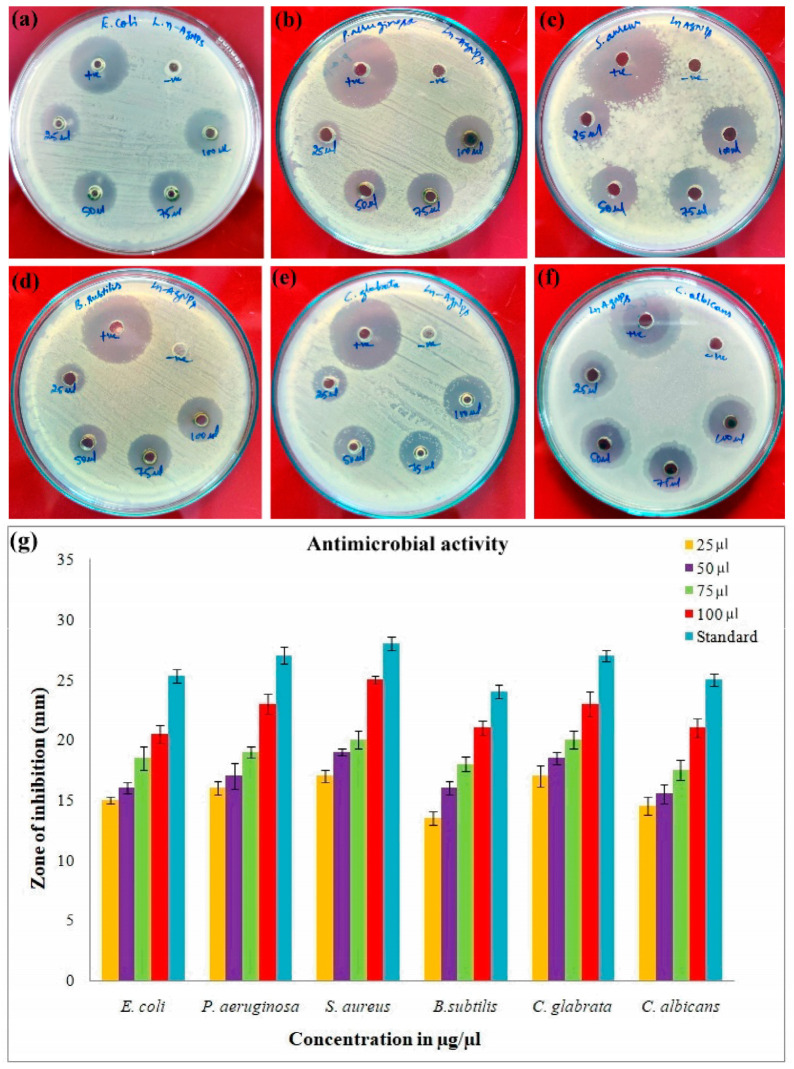
Antimicrobial activity of different concentrations of biosynthesized LnFb-AgNPs from *L. nepetifolia* flower bud extract: (**a**) *E. coli*, (**b**) *P. aeruginosa*, (**c**) *S. aureus*, (**d**) *B. subtilis*, (**e**) *C. glabrata*, and (**f**) *C. albicans*. (**g**) Graphical representation of antimicrobial activity of inhibition zones of LnFb-AgNPs against tested pathogens.

The antimicrobial efficacy of the biosynthesized LnFb-AgNPs was evaluated using the agar well diffusion method; the zone of inhibition was observed as a distinct circular zone on microbial culture plates. The LnFb-AgNPs were treated against pathogenic organisms such as Gram-positive bacteria, Gram-negative bacteria, and fungi. Our results displayed that the AgNPs capped with the *L. nepetifolia* flower bud extract exhibited maximum restriction in the growth of *P. aeruginosa*, *S. aureus*, and *C. glabrata*, with zones of inhibition measuring 23 ± 0.8 mm, 25 ± 0.2 mm, and 23 ± 1 mm, respectively, while *E. coli*, *B. subtilis*, and *C. albicans* exhibited minimal sensitivity with zones of inhibition measuring 20 ± 0.7 mm, 20.5 ± 0.5 mm, and 21.5 ± 0.7 mm when treated with a 100 µL concentration of LnFb-AgNPs. The LnFb-AgNPs treated against different pathogens are depicted in Figure 11 a–f and graphical representation for the same is displayed in Figure 11g. Global biomedical systems have recently been impacted by the emergence of pathogens with multidrug resistance. Therefore, the promising effects of silver nanoparticles on numerous pathogenic microorganisms could aid in the development of new antibacterial agents to combat pathogenic microorganisms [60]. The precise cause of AgNPs’ antibacterial sensitivity mechanism on pathogenic microorganisms is relatively well understood. Few studies have explained how AgNPs become attached to the surface of bacterial cell membranes by forming bonds with sulfur–phosphorous-containing compounds, thereby altering and interfering with the cell’s vital functions, including permeability and respiration. Thus, this leads to the degradation of enzymes, inactivation of cellular proteins, and damage of DNA. Finally, cell death occurs [35,47,61]. The smaller-sized NPs exhibited greater sensitivity against *E. coli*, *B. subtilis*, *K. pneumonia*, and *P. aeruginosa*, according to Singh (2014), who reported that dose-size-dependent AgNPs could be more effective against multidrug-resistant bacteria [62]. In a previous study, Lee et al. (2019) observed that AgNPs fabricated from a *Tussilago farfara* flower bud extract inhibited the growth of tested microbial strains in a significant manner [21].

### 3.10. In Vitro Anticancer Activity

**Figure 12:**
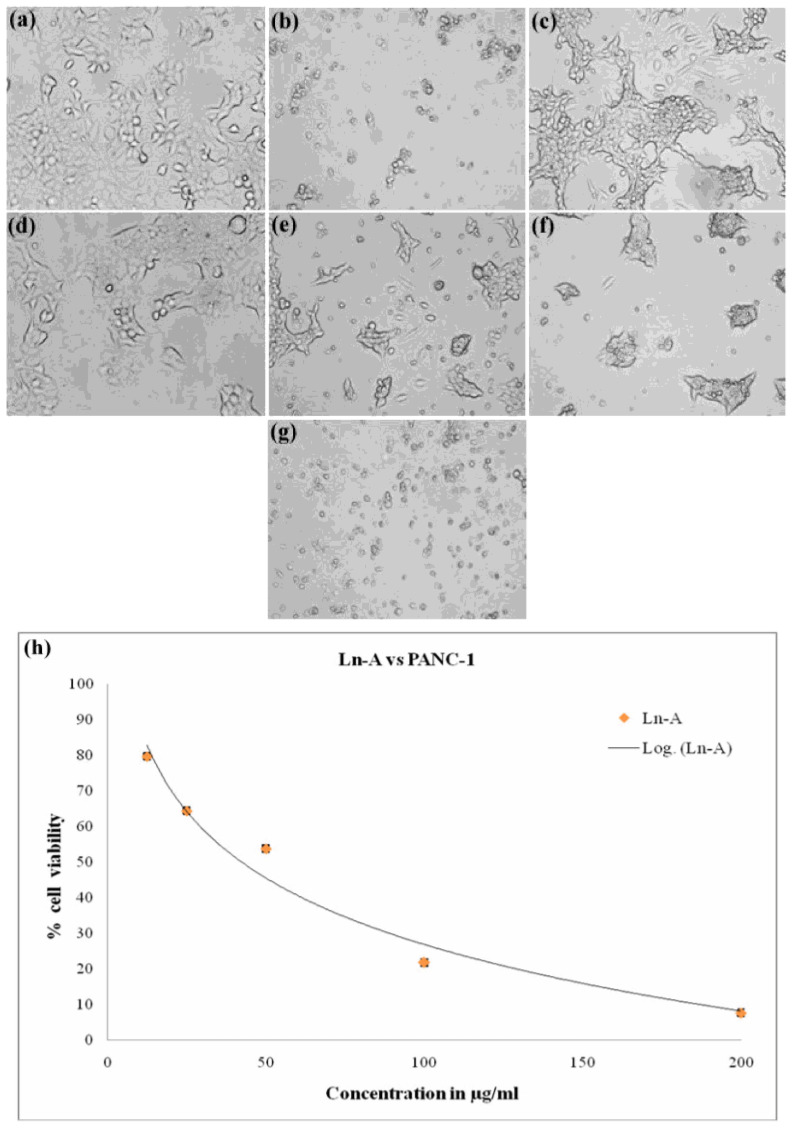
Anticancer assay of different concentration of biosynthesized LnFb-AgNPs from *L. nepetifolia* flower bud extract: (**a**) negative control, (**b**) positive control, (**c**) 12.5 μg/mL, (**d**) 25 μg/mL, (**e**) 50 μg/mL, (**f**) 100 μg/mL, and (**g**) 200 μg/mL. (**h**) Graphical representation showing comparative % of cell viability.

**Figure 13:**
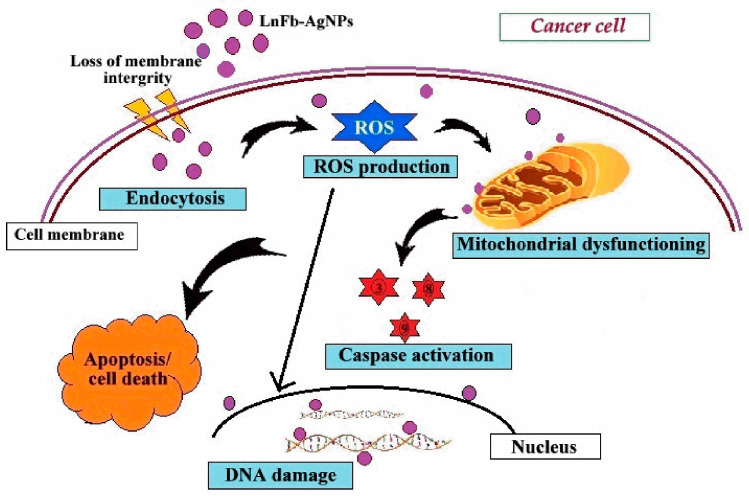
Diagrammatic representation of possible model mechanism of anticancer activity for biosynthesized LnFb-AgNPs from *L. nepetifolia* flower bud extract.

Biosynthesized LnFb-AgNPs were tested for their in vitro cytotoxicity against PANC-1 (pancreatic ductal adenocarcinoma) cells using the MTT assay. In this technique, the yellow-colored dye MTT (3-(4,5 dimethyithiazol2-yl)-2,5-diphenyltetrazolium bromide) is diminished by the mitochondrial enzyme succinate dehydrogenase, resulting in the formation of formazan (bluish-purple-colored) crystals. Since the assay is a colorimetric assessment, the results were recorded in absorbance at 570 nm. After 24 h of incubation with LnFb-AgNPs, there was a dose-dependent decrease in the relative cell viability (%) of PANC-1 cells. As the concentration of LnFb-AgNPs increased from 12.5 μg/mL to 200 μg/mL, cell viability percentages decreased proportionally. Figure 12a–g illustrate the morphological changes of cells treated with LnFb-AgNPs and positive and negative controls. Post incubation, the viability of cancer cells was 72.01%, 64.41%, 46.23%, and 21.40% at 12.5, 25, 50, and 100 μg/mL of LnFb-AgNPs, respectively, and it was reduced to a cell viability of 1.95% when treated with 200 μg/mL of AgNPs. Using the dose-dependent curve (Figure 12h), the IC_50_ value was calculated as 35.84 μg/mL. Kanniah et al. (2021) reported a similar finding, stating that green-synthesized AgNPs inhibit the viability of the PANC-1 cell line at concentrations ranging from 10 to 200 μg/mL [63]. Comparing our findings to those of Wang et al. (2021), the AgNPs synthesized from a *Zingiber officinale* leaf aqueous extract exhibited an IC_50_ of 295 μg/mL [23]. There are numerous reports on the anti-proliferative potential of AgNPs against various types of cancer cells. The potential mechanism underlying the cytotoxic potential of AgNPs against cancer cell lines is depicted in Figure 13. It is suggested that the cytotoxic potential of AgNPs is mostly due to oxidative stress and apoptosis via a caspase-dependent pathway, resulting in DNA damage and mitochondrial dysfunction and ultimately cell death [54,56,64]. In another finding reported by Shameli et al. (2021), it was revealed that the anticancer activity of a *D. regia* extract and AgNPs intensified with increasing extract doses and time. In comparison to cancerous cells, the *D. regia* extract and AgNPs had minimal effects on normal cells and did not inhibit normal cells. The differential sensitivity of MCF-7 and Panc-1 cancer cells and normal cells to the combination of the *D. regia* extract and AgNPs suggests that this combination is a promising candidate for cancer treatments [65]. Balkrishna et al. (2020) reported a similar finding, stating that even at the lowest concentration tested, AgNO_3_ is toxic to normal cells, whereas *Putranjiva roxburghii*-seed-extract-mediated silver nanoparticles (PJSNPs) exhibited no toxicity at the same concentration. This highlights the significance of the nanonization of AgNO_3_ to PJSNPs [66]. According to Barcinska et al. (2018) in a previously reported study, the effect of AgNPs on PANC-1 was significantly greater than on nontransformed pancreatic cells. They evaluated the contribution of oxidative and nitro-oxidative stress to AgNP-induced cytotoxicity against human pancreatic adenocarcinoma cells due to their crucial role in cancer cell death. The addition of AgNPs to PANC-1 cells increased the production of reactive oxygen species (ROS). Furthermore, this increase was more pronounced in cancer cells than in normal cells of the same tissue [67].

### 3.11. Apoptosis/Necrosis Studies

**Figure 14:**
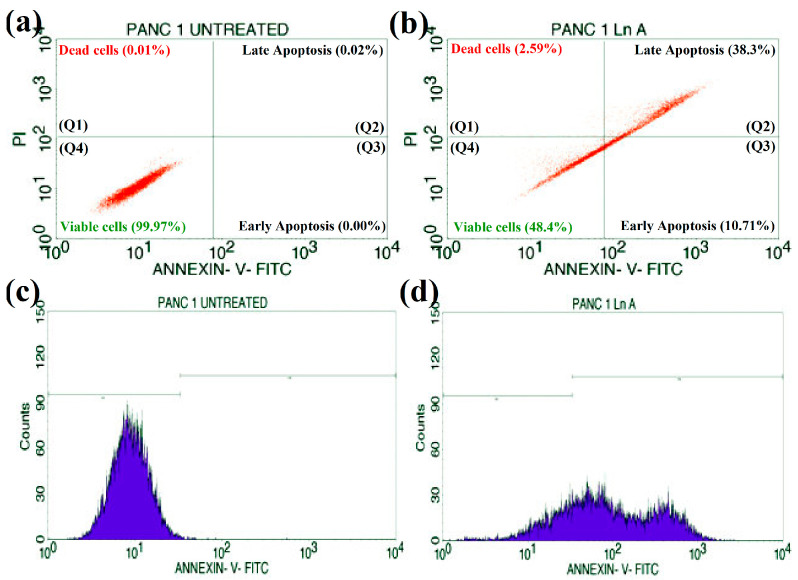
Quadrangular plot representing the Annexin V/PI expression in PANC-1 cancer cells: (**a**) untreated cells, (**b**) cells treated with LnFb-AgNPs, (**c**) cell cycle analysis of untreated cells, and (**d**) cells treated with LnFb-AgNPs analyzed by using flow cytometry.

To determine the cytotoxicity caused by silver nanoparticles capped with *L. nepetifolia* flower bud extract induced through an apoptotic pathway, Annexin V-mediated apoptosis of PANC-1 cells was studied by staining the cells with FITC Annexin V and PI, followed by fluorescence-activated cell sorting (FACS) detection flow cytometry. Flow cytometry results are displayed in Figure 14a,b. In the flow cytometry plots, the upper left quadrant (Q1) represents the percentage of dead cells (2.59%), (Q2) the upper right quadrant represents the percentage of late apoptosis (38.3%), the lower right quadrant (Q3) displays the percentage of early apoptosis (10.71%), and the last lower left quadrant (Q4) indicates the presence of viable cells percentage (48.4%). These results were obtained after 24 h of treatment with an IC_50_ concentration of 35.84 μg/mL of biosynthesized LnFb-AgNPs. The cell cycle analysis with the markers M1 and M2 is displayed in Figure 13c,d. The untreated cells expressed 99.72% of viable cells to M1 and 0.28% to M2 (Figure 14c). A total of 24.19% of viable PANC-1 cancer cells corresponded to M1, while 75.81% of damaged cells corresponded to M2 (Figure 14d). The treated cells displayed significant early and late apoptosis cell populations against PANC-1 cells, whereas untreated cells did not display any significant apoptosis. In addition, to investigate the other inhibition mechanisms, the rate of apoptosis/necrosis in the treated PANC-1 cell line was determined by an Annexin V-FITC/PI apoptosis detection assay via the flow cytometry method. Indicating the percentage of early and late apoptosis in the treated cell line, the results demonstrated that the biosynthesized LnFb-AgNPs could induce apoptosis. Similarly, Ardestani et al. (2016) reported that when AGS cancer cells were treated with 21.05 µg/mL AgNPs, the biosynthesized AgNPs exhibited 11.79% early apoptosis and 32.70% late apoptosis [54]. Ayromlou et al. (2019) also reported that the *Scorzonera calyculata* aerial part extract mediated synthesis of AgNPs showed 60% induced apoptosis against the A549 lung cancer cell line [68].

## 4. Conclusions

In the current study, an environmentally friendly, reliable, safe, and low-cost synthesis of AgNPs was accomplished using an *L. nepetifolia* flower bud aqueous extract. UV-Vis, FTIR, XRD, SEM, EDS, TEM, TGA, and Zeta potential with DLS analysis were used to characterize the phyto-synthesized LnFb-AgNPs. In UV-Vis absorption spectra, the SPR peak was detected at 418 nm. Bio-molecules were accountable for reducing and capping of silver nanoparticles, which were revealed by FTIR analysis. The XRD pattern demonstrated that the LnFb-AgNPs had a face-centered cubic crystalline structure. EDS, zeta potential, and thermo gravimetric techniques were utilized to study elemental analysis, particle stabilization, and thermal behavior. The biosynthesized LnFb-AgNPs were spherical in shape and the average particle size was 24.50 nm, and this was confirmed by TEM analysis. The DPPH free radical scavenging assay showed that the LnFb-AgNPs exhibited significantly higher antioxidant activity than the raw flower bud extract. The biosynthesized LnFb-AgNPs were found to have a pronounced antimicrobial activity towards pathogenic bacteria and fungi strains. In addition, the LnFb-AgNPs exhibited a potent cytotoxic effect against the PANC-1 cancer cell line with an IC_50_ value of 35.84 μg/mL. In addition, the apoptosis/necrosis of cancer cells was assessed using the flow cytometry method. The PANC-1 cell line exhibited significant early (10.71% of the cell population) and late (38.3% of the cell population) apoptosis. As an outcome, this research will have a significant impact on the development of improved AgNP products for the pharmaceutical, biotechnological, biomedical, and nanotechnology industries, as well as the identification of advanced drugs to treat the problem of tumor-causing cancer cells using green nanotechnology. Hence, it was suggested that the *L. nepetifolia* flower bud extract mediated synthesis of AgNPs could be used in treating pancreatic cancer after further in vivo studies.

## Data Availability

All data generated or analyzed during this study are included in this published article.
